# De Novo Sequencing of Top-Down Tandem Mass Spectra: A Next Step towards Retrieving a Complete Protein Sequence

**DOI:** 10.3390/proteomes5010006

**Published:** 2017-02-08

**Authors:** Kira Vyatkina

**Affiliations:** 1 Center for Algorithmic Biotechnology, Institute of Translational Biomedicine, Saint Petersburg State University, 7-9 Universitetskaya nab., St. Petersburg 199034, Russia; k.vyatkina@spbu.ru or vyatkina@spbau.ru; Tel.: +7-812-363-6042; 2 Department of Mathematical and Information Technologies, Saint Petersburg Academic University, 8/3 Khlopina st., St. Petersburg 194021, Russia

**Keywords:** top-down mass spectrometry, de novo sequencing, tag convolution

## Abstract

De novo sequencing of tandem (MS/MS) mass spectra represents the only way to determine the sequence of proteins from organisms with unknown genomes, or the ones not directly inscribed in a genome—such as antibodies, or novel splice variants. Top-down mass spectrometry provides new opportunities for analyzing such proteins; however, retrieving a complete protein sequence from top-down MS/MS spectra still remains a distant goal. In this paper, we review the state-of-the-art on this subject, and enhance our previously developed Twister algorithm for de novo sequencing of peptides from top-down MS/MS spectra to derive longer sequence fragments of a target protein.

## 1. Introduction

De novo sequencing of peptides and proteins from tandem (MS/MS) mass spectrometry data is an important and challenging problem, which has been attracting the attention of specialists in the field for a few decades. Most of the effort has been invested in retrieving target peptide sequences from bottom-up MS/MS data, leading to several handy software tools such as PEAKS [1], PepNovo [2], pNovo [3], Lutefisk [4], Sherenga [5], Vonode [6], Novor [7], the ALPS system [8], and a special-purpose program UVnovo [9], as well as a few alternative strategies that benefit from multiple enzyme digest [10,11,12,13,14], or pairs [15,16,17,18,19] or triples [20] of spectra acquired using different fragmentation techniques. Despite those achievements, database search is commonly considered as a substantially more reliable approach to protein identification, and remains the choice of preference if a database is available; the most widely-used tools to this end in the bottom-up and top-down case are Sequest [21] and Mascot [22], and ProSightPC/ProSight PTM [23,24] and MS-Align+ [25], respectively. However, the de novo strategy represents the only option for sequencing complementarity determining regions (CDRs) of antibodies, proteins from organisms with unknown genomes, and novel splice variants.

Top-down mass spectrometry opened new horizons in the analysis of intact proteins, particularly antibodies [26,27,28], but the number of algorithmic solutions developed for processing this kind of data still remains very limited. Until the last year, the only method for de novo sequencing of proteins solely from top-down MS/MS data was the one by Horn et al. [29] capitalizing on the complementarity of collisionally activated dissociation (CAD) and electron capture dissociation (ECD), which has never become publicly available as a software program. The next algorithm, somehow profiting from top-down MS/MS spectra, was TBNovo [30], which exploited those as a scaffold to assemble overlapping peptides reconstructed from bottom-up data. Very recently, the Twister approach [31,32], which allows for the retrieval of long and highly accurate sequence fragments of the target protein(s) from a set of top-down MS/MS spectra, has been presented and implemented in a software tool freely available on the web.

In this work, we apply the concept of *tag convolution* introduced in [33] for the case of bottom-up MS/MS data to develop a method for combining sequence fragments of the proteins from the sample into even longer, possibly gapped, amino acid sequences matching those of the proteins being analyzed. Its performance is illustrated on top-down data sets for carbonic anhydrase 2 (CAH2) and the Fab region of alemtuzumab; the sequence fragments passed to it as input comprise the amino acid sequences of the aggregated paths generated by Twister from the respective data set. The corresponding extended version of the Twister software tool can be downloaded from http://bioinf.spbau.ru/en/twister.

## 2. Results

We benchmarked the proposed approach on top-down data sets for CAH2 and alemtuzumab (see Section 4.1) using the following values of the parameters introduced in Section 4.6:

- tag length: k=4;
- maximum gap size: $G_{\max}=3000$ Da;
- minimum multiplicity of a binned distance: $B_{\min}=20$;
- minimum number of amino acids supporting a reliable binned distance (see Section 4.6): $A_{\min}=6$;
- tolerance for comparing mass offsets: $\varepsilon_{abs}=10$ ppm.

The input amino acid strings represented the sequences of the aggregated paths generated from those data sets by Twister as described in Section 4.3 and Section 4.4; further details can be found in [31,32]. In total, 70 and 92 strings were obtained for the CAH2 and alemtuzumab data set, respectively; the lists of those are provided in the supplementary file Aggregated-strings-Twister.xls. Note that some strings were due to contaminant proteins from the respective samples.

**Figure 1:**
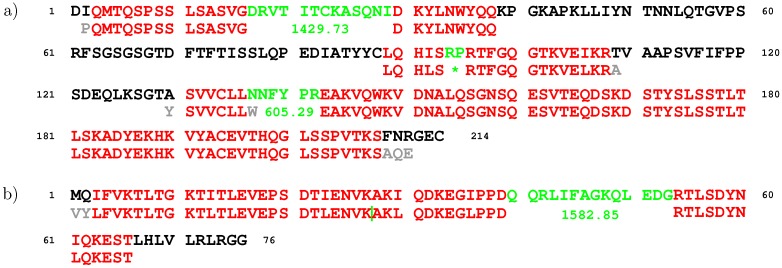
The alignment of the gapped strings against the respective protein sequence: (**a**) for the light chain of alemtuzumab; (**b**) for ubiquitin (a contaminant from the CAH2 sample). The matching fragments (up to the I/L substitution) of the gapped strings and protein sequences are marked in red; the spurious amino acids of the gapped strings are shown in gray. The gap sizes (in Da), along with the corresponding fragments of the protein sequences, are depicted in green. The zero-size gap between two adjacent fragments of ubiquitin that appeared as part of the input is indicated with the green vertical bar. The gap in the second gapped string for the light chain of alemtuzumab, labeled with the green asterisk, comprises 253.15 Da, and thus, approximately equals the mass of its corresponding dimer RP. All the other gaps accurately match the respective theoretical estimates as well.

Upon the processing of the input amino acid sequences, three and five gapped strings were formed for the CAH2 and alemtuzumab data set, respectively; see the supplementary file Gapped-strings.xls. The correct (up to the substitution I/L) sequence fragments, of at least four in length, are highlighted in color. The positions in the protein sequence of the first and last correct amino acids are provided in the fields “from” and “to”; if the former exceeds the latter, the gapped string matches the respective protein sequence in reverse. For each gap, its theoretical value is indicated; the latter is computed taking into account spurious amino acids immediately before and/or after the gap (if any). For example, the second gapped string for alemtuzumab, which corresponds to the heavy chain of the latter, contains a single gap followed by a spurious dimer GY, while the subsequence of the heavy chain of alemtuzumab that separates the respective two correct fragments is PSQT. Consequently, the theoretical estimate on the gap is calculated as Mass(PSQT)−Mass(GY). The alignment of the gapped strings corresponding to the light chain of alemtuzumab or ubiquitin against the respective protein sequence is provided in Figure 1.

The input strings grouped together always corresponded to the same protein. Out of the eight gapped strings, seven appropriately mapped to the respective protein sequence. The only exception was the fourth gapped string for alemtuzumab, which appeared due to a fully correct (up to reversal) sequence fragment PVGTQLNNTNYLLL and a spurious prolongation SQTMENLQTGV of its reversed 6-mer VGTQLN (in the respective gapped string, the latter input string occurs in the reversed form). In this case, the value of 365.3568 showed up in the output of tag convolution for the corresponding strings with a convincingly high multiplicity due to correlation between the correct tags defined by *b*- and *y*-ions, respectively (as well as their counterparts brought forth through peak reflection during preprocessing of the deconvoluted input spectra), which supported the fragment NLQTGV of the light chain of alemtuzumab. Consequently, it was interpreted as the gap estimate.

For all the other gapped strings, the reported estimates on the gaps accurately approximate the respective theoretical values, except for the second gap in the gapped string for CAH2 corresponding to ubiquitin (a contamination in the CAH2 data set): in this case, the estimate 1582.8486 is roughly 1 Da below the theoretical gap of 1583.842 Da. This discrepancy should be attributed to a ±1 Da error introduced at the time of deconvolution. The estimate on the first gap in this string is nearly zero, which appropriately reflects the fact that its first and second fragment are adjacent in the sequence of ubiquitin. The portion of this gapped string between the first and last correct amino acid spreads from the third to 66th position in the ubiquitin sequence, thus covering 63 out of 76 (82.9%) of its amino acids (see Figure 1b), which points to the ability of the method to almost fully reconstruct the sequence of a small protein.

## 3. Discussion

We have proposed a method for combining sequence fragments of proteins from the sample being analyzed into their longer subsequences containing gaps, for each of which, an accurate estimate is reported. The approach is based on the concept of tag convolution recently introduced in [33] for the case of high-resolution bottom-up MS/MS spectra. The performance of the algorithm was illustrated on the top-down data sets for CAH2 and the Fab region of alemtuzumab; the input sequence fragments comprised the amino acid strings of the aggregated paths generated from the respective data set using Twister [31,32]. In total, eight gapped string were obtained, out of which only one was incorrect. The only error was due to a simultaneous presence in the input of the correct sequence fragment PVGTQLNNTNYLLL and its incorrect alternative VGTQLNEMTQS; the latter appeared as an erroneous prolongation of the 6-mer VGTQLN of the former at the time of construction of the aggregated strings by Twister. However, this kind of spurious output can be easily recognized through visual inspection of the gapped strings produced by the algorithm.

It should be possible to further reduce, and probably eliminate, the remaining gaps through aligning the input spectra against the obtained sequences and more thoroughly examining their parts matched to the gaps. From this point of view, using the aggregated paths generated by Twister as input is clearly beneficial, since the alignment of the underlying spectra against those is naturally obtained in the process of their generation.

Consequently, the algorithm for constructing gapped sequences from the aggregated paths was implemented within Twister, the extended version of which is freely available online. Development of a method for closing the gaps in those is an essential follow-up task that we intend to address in our future research.

## 4. Materials and Methods

### 4.1. Data Sets

The computational experiments were carried out on the top-down datasets for CAH2 and the Fab region of alemtuzumab published in [31] and are available at http://bioinf.spbau.ru/en/twister. In brief, intact CAH2 was analyzed by a reversed-phase liquid chromatography (RPLC) system coupled online with a Thermo LTQ Orbitrap Elite; MS and MS/MS spectra were collected at a resolution of 240 k and 120 k, respectively. The CAH2 data set consisted of 3031 ETD, 3363 CID and 3437 HCD top-down MS/MS spectra. Alemtuzumab was digested with papain, and subsequently reduced and analyzed by RPLC coupled online with a Thermo LTQ Orbitrap Velos; MS and MS/MS spectra were acquired at a resolution of 100 k and 60 k, respectively. The data set for the Fab region of alemtuzumab comprised 4962 ETD and 4931 HCD top-down MS/MS spectra.

### 4.2. Deconvolution

The input top-down MS/MS spectra were centroided and converted to mzXML format with ReAdW, and then deisotoped and charge state deconvoluted using MS-Deconv [34] with the default parameters: maximum charge state: 30; maximum monoisotopic mass of fragment ions: 49,000 Da; signal-to-noise ratio: 1; envelopes of precursor ions were deconvoluted to derive the precursor masses of MS/MS spectra.

### 4.3. Tags

A tag of length *k*, or *k*-tag, is defined by k+1 peaks $p_{1},\ldots ,p_{k+1}$ from a spectrum *S*, such that each two neighbor ones are separated by the mass of an amino acid. Thus, a *k*-tag *t* has an amino acid sequence $s\left(t\right)=a_{1}\ldots a_{k}$ and an offset o(t) equal to the mass $Mass(p_{1})$ of the leftmost peak $p_{1}$.

A set T of 4-tags, to become the input for tag convolution, was generated with the method implemented within the Twister software tool [31,32] for de novo sequencing of peptides from top-down tandem mass spectra. Thereby, the default parameters of Twister were used: tag length k=4, mass tolerance ε=4 mDa, peak reflection applied to individual deconvoluted spectra, and water loss ions eliminated. Further, for a preprocessed spectrum *S*, a spectrum graph G(S) was constructed, the vertices of which corresponded to the peaks of *S*, and for two vertices—*u* and *w*—an edge from *u* to *w* was introduced if m(w)−m(u) matched the mass of some amino acid within 2ε, where m(v) denotes the mass of the peak from *S* that gave rise to the vertex *v*. The vertices of G(S) were scored with the intensities of their underlying peaks, and an optimal path with respect to the vertex scores was extracted from each connected component of G(s). Finally, from each obtained path of at least k=4 in length, all the possible 4-tags were derived.

An important point here is that the application of a small constant mass tolerance at the time of generating the edges of G(S) assures that the resulting *k*-tags are highly accurate. A detailed description of the above procedure can be found in [31].

### 4.4. Sequence Fragments

The first part of the input of the proposed method is a set A of amino acid strings supposed to represent sequence fragments of the proteins from the sample being analyzed. In our experiments, we used as A the amino acid sequences of the aggregated paths generated with Twister, as described in [32], from the set of MS/MS spectra acquired from the respective sample.

In brief, Twister takes a set of deisotoped and charge state deconvoluted MS/MS spectra as input, and first generates from them a set of highly accurate *k*-tags using the strategy described in the previous section. Next, it assembles a number of de novo strings from the tags consistent with each other in terms of both amino acid sequences and offsets, each assigned a mass offset equal to the smallest offset among those of the tags contributing to it. (For example, if we have two 4-tags derived from HCD spectra, with the amino acid string SGAT and GATF, respectively, and offset 500 and 587, respectively, we note that 587=500+Mass(S), and therefore, those tags may be due to the same protein—e.g., one with a subsequence SGATF preceded by an N-terminal fragment of mass 500; having glued the two tags, we will obtain a de novo string SGATF with the offset of 500.) Finally, Twister combines the derived de novo strings into a number of *aggregated strings* endowed with direct and reversed offsets; the amino acid sequence of an aggregated string typically represents a longer sequence fragment of a protein contained in the sample, and its associated offsets reflect the location of the respective fragment within the entire sequence.

To generate the aggregated strings, we ran Twister with the default parameters (see above) on the CAH2 and alemtuzumab data sets. The amino acid sequences of the 70 and 92 aggregated strings obtained for CAH2 and alemtuzumab, respectively, which served as input for the algorithm being described, are listed in the supplementary file Aggregated-strings-Twister.xls. Their correct fragments, at least four in length, are highlighted in color, and for each of those, its first and last position in the corresponding protein sequence is indicated; in the case that the former exceeds the latter, the respective fragment occurs in the sequence in the reversed form.

### 4.5. Tag Convolution

For an amino acid sequence *s*, let $\bar{s}$ denote its reversed copy.

Tag convolution was defined in [33] as follows. For a set of *k*-tags T, let K(T)={w|∃t∈T:s(t)=w} denote the set of all their amino acid sequences. Given two *k*-mers $w_{1},w_{2}\in K\left(T\right)$, tag convolution $\tau (w_{1},w_{2})$ considers all pairs $\left(t_{1},t_{2}\right)$ of tags from T, such that $s\left(t_{1}\right)=w_{1}$ and $s\left(t_{2}\right)=w_{2}$, and computes the difference $o\left(t_{2}\right)-o\left(t_{1}\right)$ of their offsets. For each difference encountered thereby (up to a predefined tolerance), tag convolution records how many times it occurred. Thus, its output comprises a set of pairs, each composed of a registered offset difference $d_{i}$ and its multiplicity $m_{i}$: $\tau \left(w_{1},w_{2}\right)=\left\{\left(d_{i},m_{i}\right)|1\leq i\leq h\right\}$, where *h* is the number of distinct offset difference values observed.

Subsequently, the above concept was generalized to the case of strings, and slightly adjusted so that for two subsequences $s_{1}=a_{i}\ldots a_{i+q}$ and $s_{2}=a_{j}\ldots a_{j+r}$ of *s*, where 1≤i≤i+q<j≤n−r, the value contributed to the output of tag convolution $T(s_{1},s_{2})$ by the pairs of tags matching either $s_{1}$ and $s_{2}$ or $\bar{s_{2}}$ and $\bar{s_{1}}$ would equal $Mass(a_{i+q+1}\ldots a_{j-1})$, i.e., the mass of the subsequence separating $s_{1}$ and $s_{2}$ in *s*. This was formalized in the following way.

For a real *δ*, a shift of $\tau (w_{1},w_{2})$ by *δ* is defined as $\tau_{\delta }\left(w_{1},w_{2}\right)=\left\{\left(d+\delta ,m\right)|\left(d,m\right)\in \tau \left(w_{1},w_{2}\right)\right\}$. To compute $T(s_{1},s_{2})$ for two amino acid strings $s_{1}=x_{1}\ldots x_{e}$ and $s_{2}=y_{1}\ldots y_{f}$, we first iterate over all the pairs of *k*-mers from $s_{1}$ and $s_{2}$, respectively; thereby, a pair $\left(x_{i}\ldots x_{i+k-1},y_{j}\ldots y_{j+k-1}\right)$ contributes the output of $\tau_{-Mass\left(x_{i}\ldots x_{e}\right)-Mass\left(y_{1}\ldots y_{j-1}\right)}\left(x_{i}\ldots x_{i+k-1},y_{j}\ldots y_{j+k-1}\right)$ to an auxiliary set $\tau (s_{1},s_{2})$. Next, we analogously form a set $\tau (\bar{s_{2}},\bar{s_{1}})$. Having merged together $\tau (s_{1},s_{2})$ and $\tau (\bar{s_{2}},\bar{s_{1}})$, we obtain $T(s_{1},s_{2})$. Note that $T\left(s_{1},s_{2}\right)=T\left(\bar{s_{2}},\bar{s_{1}}\right)$.

In [33], we described a procedure for validating de novo peptide sequences. In particular, for an amino acid $a_{i}$ of a candidate sequence $s=a_{1}\ldots a_{n}$, where k<i≤n−k, it computes $T(a_{1}\ldots a_{i-1},a_{i+1}\ldots a_{n})$ and checks whether $Mass(a_{i})$ occurs in it with a high enough multiplicity. According to our experiments, for a correct peptide sequence *s*, the multiplicity $Mass(a_{i})$ usually clearly dominates that of the other values present in $T(a_{1}\ldots a_{i-1},a_{i+1}\ldots a_{n})$. This suggests that a similar idea might be applied to check whether two amino acid strings $s_{1}$ and $s_{2}$ are subsequences of a longer sequence *s*: to this end, one would compute $T(s_{1},s_{2})$ and verify whether the multiplicity of the most frequently observed offset difference $d^{*}$ is significantly greater than the second-highest multiplicity. If so, $d^{*}$ would be reported as the mass of the subsequence separating $s_{1}$ and $s_{2}$ in *s*; otherwise, the verdict would be that $s_{1}$, $s_{2}$ and *s* are not related in that way.

However, such an approach would work fine only for a rather short peptide sequence *s*, and its subsequences $s_{1}$ and $s_{2}$ separated by at most a few amino acids, and turns out to be inapplicable to the top-down case, with long protein sequences and large gaps between the retrieved fragments of those. The underlying issues, along with the means to resolve them, are discussed in the next section.

### 4.6. Gap Estimation

Given two amino acid strings $s_{1}$ and $s_{2}$, we aim to verify whether they represent two disjoint fragments of the same protein sequence *s*, and if the answer is positive, report an approximate mass of the sequence separating them in *s*. To this end, we compute $T(s_{1},s_{2})$ based on a set T of *k*-tags extracted from top-down MS/MS spectra; however, only pairs of tags from the same spectrum are allowed to contribute to $T(s_{1},s_{2})$, and its output needs to be treated in a different way, as compared to the bottom-up case.

To generate the set T, we again apply the strategy being part of the Twister approach, which assures high accuracy of the resulting tags (see Section 4.3). In particular, we use a stringent mass tolerance ε=4 mDa when deciding whether the difference between two peak masses matches the mass of some amino acid, thereby relying upon the observation that the errors in close masses tend to be similar.

However, when we switch to the differences between tag offsets, which can be quite large, this kind of assumption can no longer be made. Moreover, the same value can appear as a difference of two relatively small offsets, and also as that of two large offsets, and in the latter case, the error in it may be substantially larger than in the former case. To avoid the need to keep track of the way in which concrete values were obtained, we apply the binning strategy similar to the one introduced in [32] for analyzing the offsets of aggregated strings. Furthermore, namely, each offset difference *d* is first scaled through multiplication by $10^{h}$ (in our experiments, h=4), and rounded to the nearest integer; subsequently, each obtained *scaled difference* $d^{s}$ is assigned a multiplicity $\mu (d^{s})$ equal to the number of the offset differences that got transformed into it. In addition, an integral *binned difference* $d^{b}$ is calculated for *d* by rounding it to the nearest integer; its multiplicity is defined as $\mu \left(d^{b}\right)=\mu \left(d_{1}^{s}\right)+\ldots +\mu \left(d_{g}^{s}\right)$, where $d_{i}^{s}$ are the scaled counterparts of the offset differences that got transformed into $d^{b}$, 1≤i≤g.

Let our hypothesis be that $s_{1}$ and $s_{2}$ are two disjoint subsequences of the same (unknown) protein sequence *s*, and $s_{1}$ precedes $s_{2}$ in *s*. In order to disprove it, we proceed as follows. First, we calculate $T(s_{1},s_{2})$, along with the respective sets of scaled and binned offset differences endowed with multiplicities. Next, we focus on the binned differences, and select the non-negative ones not exceeding a predefined threshold $G_{\max}$. Further, from the binned differences still under consideration, which have the multiplicity at least $B_{\min}$, we pick up those with the highest multiplicity $b^{\max}$. For each such difference $d^{b_{\max}}$, we calculate its score as $Score\left(d^{b_{\max}}\right)=\mu \left(d^{b_{\max}}\right)+\mu \left(d^{b_{\max}}-1\right)+\mu \left(d^{b_{\max}}+1\right)$, assuming that a value $d^{\prime }$ that does not appear as a binned difference has a zero multiplicity. In this way, we account for the well-known ±1 Da errors in large enough deconvoluted masses. Finally, the top-scoring binned difference $d_{top}^{b_{\max}}$ is selected (the smallest one is picked up in case of ties), then its corresponding scaled difference $d_{0}^{s}$ with the highest multiplicity is detected, and the value of $\hat{d}=d_{0}^{s}\cdot 10^{-h}$ is reported as a candidate estimate of the gap between $s_{1}$ and $s_{2}$.

As a last step, we check whether the tags that contributed to the binned counterpart ${\hat{d}}^{b}$ of the estimate $\hat{d}$ together would cover at least a certain number of amino acids in both $s_{1}$ and $s_{2}$. To this end, we introduce a threshold $A_{\min}$, and note that $m^{*}=A_{\min}-k+1$ *k*-tags with distinct labels all corresponding to the same string will always cover $A_{\min}$ amino acids in it. The estimate $\hat{d}$ is accepted if at least $m^{*}$ tags that support ${\hat{d}}^{b}$ are observed for each of $s_{1}$ and $s_{2}$, or $m^{*}+1$ and $m^{*}-1$ tags are observed for one and the other string, respectively. If neither is the case, we check whether the respective numbers are both at least $m^{*}-1$, and if so, whether either ${\hat{d}}^{b}-1$ or ${\hat{d}}^{b}+1$ occurred among the binned differences, and was supported by at least $m^{*}-1$ and $m^{*}$ tags for the two strings, respectively. In case this holds, the estimate $\hat{d}$ is accepted. Otherwise, we conclude that the hypothesis was wrong.

Since the protein sequence fragments may appear in the output of Twister in a direct as well as reversed form, when processing the amino acid sequences $s_{1}$ and $s_{2}$ of two aggregated paths, we apply the above procedure to up to four pairs of strings, and namely, $s_{1}$ and $s_{2}$, $s_{1}$ and $\bar{s_{2}}$, $\bar{s_{1}}$ and $s_{2}$, and $\bar{s_{1}}$ and $\bar{s_{2}}$. If for some pair, a gap estimate was obtained, the two strings are joined to form a gapped path, and the remaining pairs are not considered.

To enable iterative construction of gapped paths, we proceed as follows. The gapped paths are initialized with the input strings, and further examined pairwise. As in the case of regular amino acid strings, for a pair $g_{1}$, $g_{2}$ of gapped paths, we consider four combinations comprising the direct and/or reversed versions of those: $g_{1}$ and $g_{2}$, $g_{1}$ and $\bar{g_{2}}$, $\bar{g_{1}}$ and $g_{2}$, and $\bar{g_{1}}$ and $\bar{g_{2}}$. Without loss of generality, let us discuss in more detail the first case.

When processing $g_{1}$ and $g_{2}$, we first try to append $g_{2}$ to $g_{1}$. To decide whether it is possible, we pick up the last sequence fragment $s_{1}^{last}$ of $g_{1}$ and the first sequence fragment $s_{2}^{first}$ of $g_{2}$, and verify as stated above whether $s_{1}^{last}$ and $s_{2}^{first}$ represent two fragments of the same protein sequence. If the answer is positive, $g_{2}$ is appended to $g_{1}$; otherwise, we consecutively examine the gaps from $g_{1}$, and for each gap large enough to potentially accommodate $g_{2}$, perform a similar check for the sequence fragment $s^{\prime }$ of $g_{1}$ immediately preceding this gap, and $s_{2}^{first}$. If, according to its outcome, $s^{\prime }$ precedes $s_{2}^{first}$ in some protein sequence, we additionally verify whether upon embedding of $g_{2}$ into this gap, its tail would overlap the fragment $s^{\prime \prime }$ of $g_{1}$ immediately after the gap. If not, $g_{2}$ is appropriately merged into $g_{1}$ after $s^{\prime }$. The overlap check amounts to a comparison of the mass offset of the end of $g_{2}$ upon embedding, and that of the beginning of $s^{\prime \prime }$ (the offsets may be calculated e.g., with respect to the beginning of $g_{1}$), which is carried out using a tolerance $\varepsilon_{abs}$ specified in ppm. In case $g_{2}$ could not be embedded into $g_{1}$, a similar procedure is applied with a goal of embedding $g_{1}$ into $g_{2}$.

## Supplementary Materials

The following are available online at www.mdpi.com/2227-7382/5/1/6/s1: File Aggregated-strings-Twister.xls containing the lists of the amino acid sequences of the aggregated strings generated by Twister from the data sets for CAH2 and alemtuzumab, respectively; File Gapped-strings.xls containing the lists of the gapped strings obtained for CAH2 and alemtuzumab, respectively.

## Abbreviations

The following abbreviations are used in this manuscript:

MS/MS	tandem mass spectrometry
CAD	collisionally activated dissociation
ECD	electron capture dissociation
CAH2	carbonic anhydrase 2
CDR	complementarity determining region
RPLC	reversed-phase liquid chromatography
MS	mass spectrometry
ETD	electron-transfer dissociation
CID	collision-induced dissociation
HCD	higher-energy C-trap dissociation
